# Donepezil ameliorates oxygen-glucose deprivation/reoxygenation-induced brain microvascular endothelial cell dysfunction via the SIRT1/FOXO3a/NF-κB pathways

**DOI:** 10.1080/21655979.2022.2045833

**Published:** 2022-03-14

**Authors:** Xueming Sun, Bing Liu

**Affiliations:** Baotou Vocational and Technical College, Baotou City, Inner Mongolia, China

**Keywords:** Donepezil, OGD/R, ischemic stroke, human brain microvascular endothelial cells, SIRT1

## Abstract

Ischemic stroke is a disease in which brain tissue is damaged by a sudden rupture or blockage of a blood vessel in the brain that prevents blood from flowing to the brain. Extensive literature has demonstrated the neuroprotective effect of donepezil on brain injury, and this paper attempts to further reveal the effect of donepezil on brain microvascular endothelial cells dysfunction. Human brain microvascular endothelial cells (HBMECs) were treated with oxygen-glucose deprivation/reoxygenation (OGD/R) to induced brain microvascular endothelial cell dysfunction. The OGD/R-induced cell were added with different doses of donepezil with or without Sirtuin-1 (SIRT1) inhibitor EX527. Cell viability of HBMECs was examined by cell counting kit (CCK)-8 assay. OGD/R-treated cell migration was assessed by wound healing assay while angiogenesis in HBMECs was examined by tube formation assay and Western blot analysis. Endothelial cell dysfunction was assessed employing fluorescein isothiocyanate-dextran assay and Western blotting. SIRTI/FOXO3a/NF-kB signaling pathway-related protein expressions were detected using Western blotting. After pretreatment with SIRT1 inhibitor EX527, the above experiments were done again. Donepezil increased cell viability of OGD/R-induced HBMECs, promoted cell migration and angiogenesis, decreased cell permeability, and upregulated the expressions of tight junction proteins. In addition, donepezil regulated the expressions of SIRT1/FOXO3a/NF-κB signaling pathways. However, pretreatment with the SIRT1 inhibitor EX527 reversed the protective effect of donepezil on OGD/R-induced HBMECs. In summary, Donepezil ameliorates OGD/R-induced brain microvascular endothelial cell dysfunction via the SIRT1/FOXO3a/NF-κB pathways.

## Graphical abstract

**Figure:**
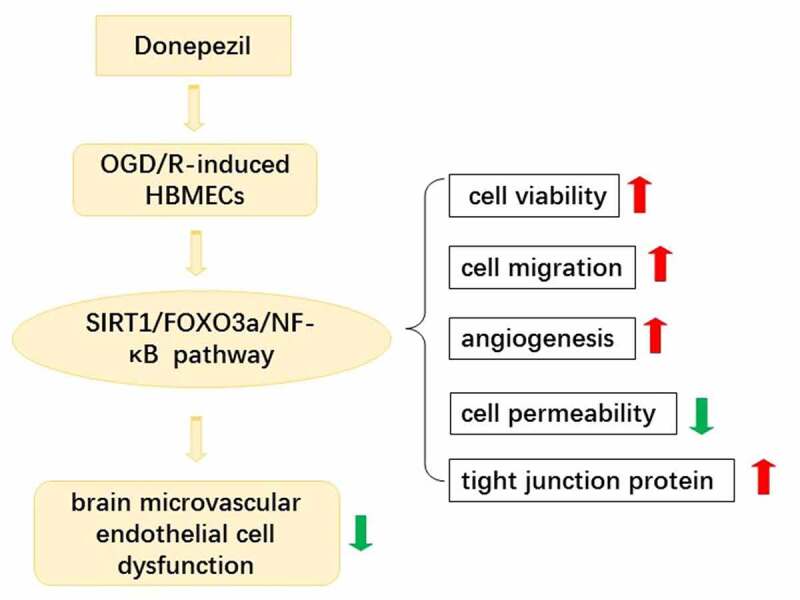

## Introduction

Ischemic stroke is the primary type of cerebrovascular disease that typically leads to death or disability in the elderly population worldwide [1,2]. Cerebral ischemia is primarily a brain injury caused by reduced or stopped blood flow to the brain and can lead to permanent neurological deficits, dementia, or death [3]. Currently, intravenous thrombolysis, endovascular thrombectomy and timely blood perfusion are the main effective treatments in the early stages of ischemic stroke [4,5]. However, the limitations of pharmacological thrombolytic therapy are the short therapeutic window, the risk of bleeding, and the possible damage of reperfusion/ischemic injury [6]. One of the key factors in the development of cerebral ischemia is the disruption of the blood-brain barrier (BBB), which can regulate the trafficking of fluid, solute and cells at the blood-brain interface, as well as keeping the central nervous system in balance [7]. BBB includes four key cells: astrocytes, pericytes, neuron and brain microvascular endothelial cells (HBMECs) [8]. Among them, HBMECs are the core factor of BBB, can constitute a unique cellular barrier to sustain brain homeostasis [9]. When the integrity of the BBB is disrupted under the occurrence of ischemic stroke, and HBMECs are the first to be affected by ischemic damage in the brain [10]. Therefore, repairing the dysfunction of HBMECs is one of the important means to ameliorate the ischemic damage in the brain.

Donepezil is a second-generation cholinesterase inhibitor approved for the treatment of dementia caused by Alzheimer’s disease [11]. Recently, there is a large body of literature addressing the neuroprotective effects of donepezil and the ameliorative effects of ischemia-reperfusion injury. For example, donepezil is neuroprotective in brain injury and Alzheimer’s disease under cardiac ischemia-reconcern injury [12]. Donepezil hydrochloride has a protective effect against ischemia/reperfusion injury in the mouse brain [13]. Donepezil attenuates injury after ischemic stroke by stimulating neurogenesis, angiogenesis, inhibition of inflammation and apoptosis [14]. In addition, donepezil has been shown to have a protective effect on endothelial cell permeability [15]. For instance, donepezil protects endothelial cells against hydrogen peroxide-induced cell injury [16]. Donepezil, tacrine and α-phenyl-n-tert-butylnitrone inhibit choline transport by conditionally immortalizing rat brain capillary endothelial cell lines [17]. However, the effect of donepezil on OGD/R-induced brain microvascular endothelial cells dysfunction has not yet been reported.

Sirtuin-1 (SIRT1) is a class-III histone deacetylase involved in gene silencing, cell cycle, cellular oxidative stress and senescence [18]. SIRT1 was reported to stimulate the transcription of FOXO3a target genes through deacetylation of FOXO3a [19]. It has been shown that paeonol inhibits high glucose and palmitic acid-induced apoptosis, oxidative stress and inflammatory responses in human umbilical vein endothelial cells via the SIRT1/FOXO3a/NF-κB pathway [20]. In addition, lncRNA Snhg8 attenuates microglia inflammatory response and blood-brain barrier damage in ischemic stroke by regulating miR-425-5p-mediated SIRT1/NF-κB signaling pathway [21]. Donepezil attenuates high glucose-accelerated senescence in human umbilical vein endothelial cells through SIRT1 activation [22]. Accordingly, in this study, we aimed to identify the effects of donepezil on OGD/R-induced HBMECs and its related mechanisms. We speculated that donepezil may have a protective effect on HBMECs through activating the SIRT1/FOXO3a/NF-κB signaling pathways.

## Materials and methods

### Cell culture and treatment

The human brain microvascular endothelial cells (HBMECs) used in this study were supplied by the cell bank of Shanghai Biology Institute (Shanghai, China). These cells were grown in Dulbecco’s Modified Eagle’s Medium (DMEM) with 10% fetal bovine serum (FBS) and 1% Penicillin/Streptomycin solution in a humidified atmosphere with 5% CO_2_ at 37°C. For the establishment of I/R conditions in vitro, HBMECs were cultured in a glucose- and serum-free DMEM placed in a hypoxic cell culture system for 2 h at 37°C, 5% CO_2_ and 95% N_2_. Then, cells were maintained in a glucose-containing DMEM with 10% FBS in an incubator with 95% air and 5% CO_2_ for 6 h. Those cultured in a complete medium only at 37°C, 5% CO_2_ were served as a control [23].

To investigate the effect of donepezil on the cells, we treated the cells with donepezil (Eisai, Suzhou, China) at concentrations of 20, 50, and 100 μM for 2 h.

### Cell counting kit (CCK)-8

The HBMECs treated with donepezil (20, 50, 100 μM) and OGD/R were grown in 96-well plates for 24 h incubation. Then, the CCK-8 assay solution (Yeasen, Shanghai, China) was added into the wells for 4 h incubation as recommended by the manufacturer. The optical density (OD = 450 nm) was checked by a microplate reader (Reagen, Shenzhen, China) [24].

### Wound healing assay

The cell migration across a scratch gap was assayed by wound healing [25]. Briefly, HBMECs were cultured in plates until confluence. The scratches was made by a 10 μL pipette tip after OGD/R treatment. Then, following washing with PBS for three times, these cells were incubated again for 24 h. After that, cell migration was qualitatively assessed by the size of the wounds at the end of the experiment using Image J software. The migration rate was calculated based on the formula: (wound width at 0 h – wound width at 24 h)/wound width at 0 h × 100%.

### Tube formation assay

A 70 µL solution of Matrigel (BD Biosciences, San Jose, CA, USA) was added to a pre-cooled 96-well plate and incubated at 37°C for 30 min. The HBMECs were seeded into the wells containing the Matrigel and cultured overnight. The tube structure was observed adopting a microscope (Olympus, Beijing, China). The total number of branches and tube length were calculated with the application of Image J software (National Institutes of Health, Maryland, USA), and each experiment was repeated at least three times [26].

### Western blot analysis

The cell lysates of HBMECs were made adopting RIPA buffer (Beyotime, Shanghai, China) with 1% protease and phosphatase inhibitor cocktail at 4°C and centrifuged. The supernatant was then collected for the protein concentration detection using Bradford Protein Assay Kit (Yubo Biological Technology, Shanghai, China). Protein in equal amounts was separated by 12% SDS-PAGE, followed by a transferring onto PVDF membranes. After blocking with 5% nonfat milk at room temperature for 1 h, the incubation of membranes was conducted with primary antibodies targeting VEGF (Beyotime, AV202, 1:1000), p-VEGFR2 (Bioworld, Y1175, 1:1000), VEGFR2 (Abcam, ab134191, 1:1000), ZO-1 (Abcam, ab216880, 1:1000), VE-cadherin (Abcam, ab205336, 1:1000), Claudin-1 (Abcam, ab211737, 1:2000), SIRT1 (Abcam, ab189494, 1:1000), FOXO3a (Abcam, ab109629, 1:1000), p-p65 (R&D, YB-22488, 1 mg/ml), p65 (Abcam, ab32536, 1:1000) overnight at 4°C. After washing with TBST for three times, the horseradish peroxidase-conjugated secondary antibody was added for 1 h incubation at room temperature. An enhanced chemiluminescence (ECL; Tanon, Shanghai, China) detection system was adopted to detect the protein bands. The quantification of protein bands were carried out with the aid of Image Lab 3.0 software (Bio-Rad, Hercules, CA, USA).

### In vitro *permeability test kit*

The endothelial permeability of HBMECs was assayed with the help of FITC-dextran [27]. First, HBMECs (1 × 10^5^) were grown in 24-well transwell and cultured at 37°C in 5% CO_2_. 48 h incubation later, the dextran (1 mg/ml) labeled fluorescently was added to the culture medium in the upper chamber for 1 h incubation. Afterward, the culture medium in the lower chamber were collected and the amount of FITC-dextran was measured at an excitation wavelength of 485 nm and an emission wavelength of 535 nm using a fluorescence plate reader (Infinite M200 Pro, Tecan Group, Mannerdorf).

### Statistical analysis

The normal distribution of data was recorded in the way of mean ± SD from at least three independent experiments and data were analyzed by GraphPad PRISM 5.0 software (San Diego, CA, USA). Significant differences between multiple groups were analyzed by one‐way analysis of variance followed by Bonferroni post hoc comparisons tests. P < 0.05 was used as an indication of a statistically significant difference.

## Results

In this study, we studied the effects of donepezil and the potential mechanism in OGD/R-induced HBMECs. The results demonstrated that the donepezil promoted the cell viability, cell migration and angiogenesis, abated cell permeability in OGD/R-induced HBMECs. In addition, donepezil mediated the SIRT1/FOXO3a/NF-κB signaling pathways. Moreover, treatment with the SIRT1 inhibitor EX527 reversed the protective effect of donepezil on OGD/R-induced HBMECs.

### Donepezil increases the cell viability of OGD/R-induced HBMECs

**Figure 1.**
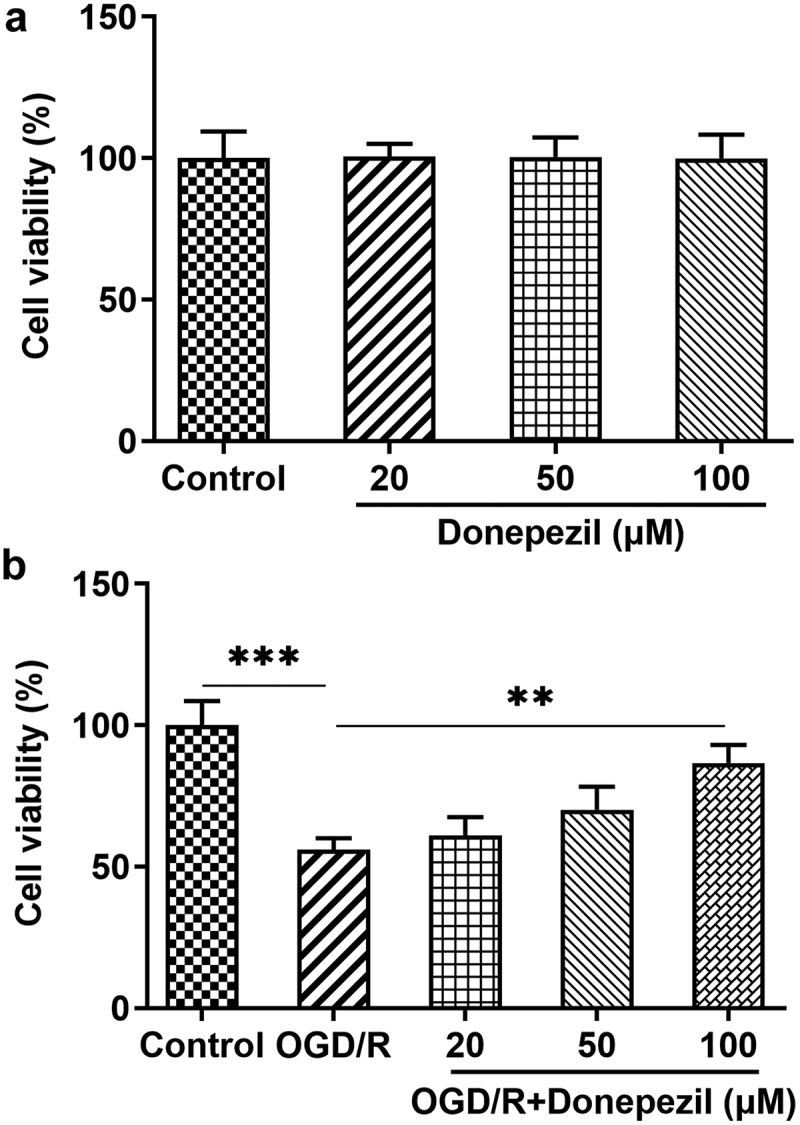
Donepezil increases the cell viability of OGD/R-induced HBMECs. (a) Cell viability of HBMECs treated with donepezil at the concentration of 20, 50, 100 μM was assessed with the help of CCK-8. (b) Cell viability of HBMECs treated with OGD/R and donepezil at the concentration of 20, 50, 100 μM was assessed with the help of CCK-8. Results are the mean ± SD. **P < 0.01, ***P < 0.001.

To assess effects of donepezil on OGD/R-induced HBMECs, the cell viability of HBMECs exposed to OGD/R was firstly detected. We performed CCK-8 assay in HBMECs treated with donepezil (20, 50, 100 μM) or OGD/R+ donepezil. It can be seen in Figure 1(a) that the viability of HBMECs remained unchanged after the treatment of donepezil at the concentration of 20, 50, 100 μM compared to the control group. Figure 1(b) presented that the viability of HBMECs decreased by around 45% in the OGD/R group (vs Control) and rose in a concentration-dependent manner in the OGD/R+ donepezil (20, 50, 100 μM) groups. Thus, increased concentration of donepezil could largely prevent OGD/R-treated loss of cell viability in HBMECs.

### Donepezil promotes OGD/R-treated cell migration and angiogenesis in HBMECs

**Figure 2.**
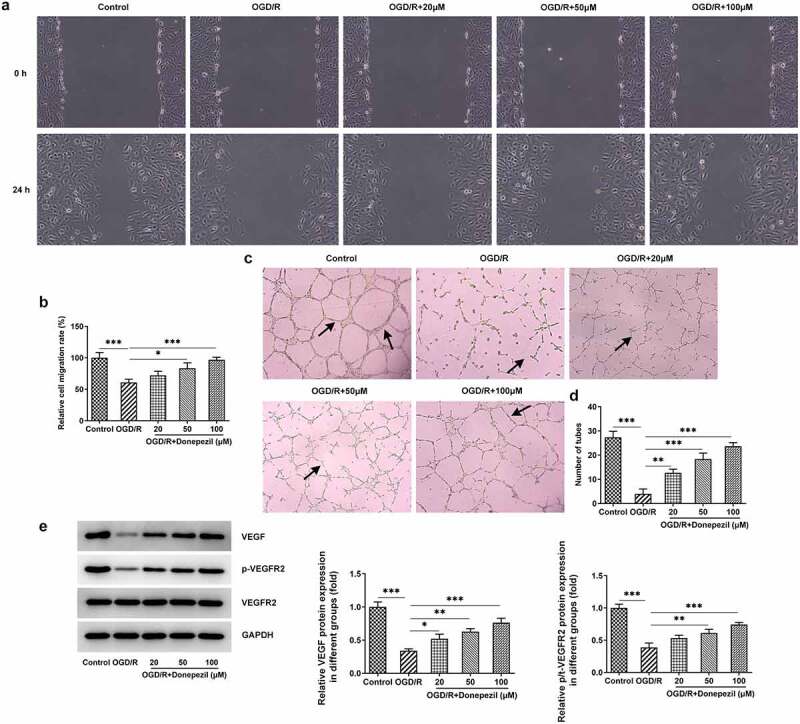
Donepezil Promotes OGD/R-treated Cell Migration and Angiogenesis in HBMECs. (a-b) The capacity of cell migration in OGD/R-induced HBMECs treated with donepezil (20, 50, 100 μM) was detected employing wound healing. (c-d) The capacity of angiogenesis in OGD/R-induced HBMECs treated with donepezil (20, 50, 100 μM) was assayed by means of tube formation. (e) The expressions of angiogenetic-related proteins VEGF, p-VEGFR2 and VEGFR2 were examined in OGD/R-induced HBMECs treated with donepezil (20, 50, 100 μM) by the way of Western blot. Results are the mean ± SD. *P < 0.05, **P < 0.01, ***P < 0.001.

Then, we examined the effects of donepezil on the ability of cell migration and angiogenesis in OGD/R-induced HBMECs by the assays of wound healing and tube formation. Figure 2(a-b) showed the lower cell migration capacity of HBMECs in the OGD/R group (vs Control) and the progressively higher cell migration capacity of HBMECs in the OGD/R+ donepezil (20, 50, 100 μM) groups. As shown in Figure 2(c-d), OGD/R induced a marked decrease in angiogenic capacity (vs Control), whereas donepezil increased the capacity of angiogenic in a concentration-dependent manner. Additionally, the expressions of angiogenesis-related proteins VEGF and p-VEGFR2 were remarkably declined in the OGD/R group in comparison with the control group, but rose steadily in the OGD/R+ donepezil groups (Figure 2(e)). Therefore, these results suggest that donepezil could promote the cell migration and angiogenesis of HBMECs induced by OGD/R.

### Donepezil decreases OGD/R-induced cell permeability and upregulates tight junction protein expression in HBMECs

**Figure 3.**
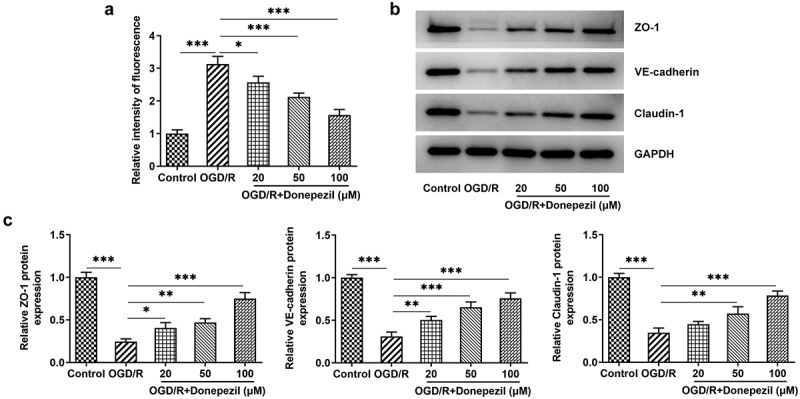
Donepezil decreases OGD/R-induced cell permeability and upregulates tight junction protein expression in HBMECs. (a) The relative fluorescence intensity of OGD/R-induced HBMECs treated with donepezil (20, 50, 100 μM) was measured with the application of *in vitro* permeability test kit. (b-c) The expressions of related proteins ZO-1, VE-cadherin and Clarudin-1 were determined in OGD/R-induced HBMECs treated with donepezil (20, 50, 100 μM) employing Western blot. Results are the mean ± SD. *P < 0.05, **P < 0.01, ***P < 0.001.

To identify the effect of donepezil on the endothelial barrier function of OGD/R-induced HBMECs cells, we tested the cell permeability and the expressions of tight junction proteins. Figure 3(a) showed that HBMECs showed a stronger fluorescence intensity in the OGD/R group (vs Control) and gradually decreasing fluorescence intensity in the OGD/R+ donepezil groups. As can be seen from Figure 3(b-c), Western blot also detected a decline in the expressions of ZO-1, VE-cadherin and Claudin-1 in the OGD/R group and an increase in the expressions of ZO-1, VE-cadherin and Claudin-1 in the OGD/R+ donepezil groups. Overall, these results indicate that donepezil could reduce cell permeability and upregulate the expressions of tight junction proteins in OGD/R-induced HBMECs.

### Donepezil regulates the SIRT1/FOXO3a/NF-κB signaling pathway

**Figure 4.**
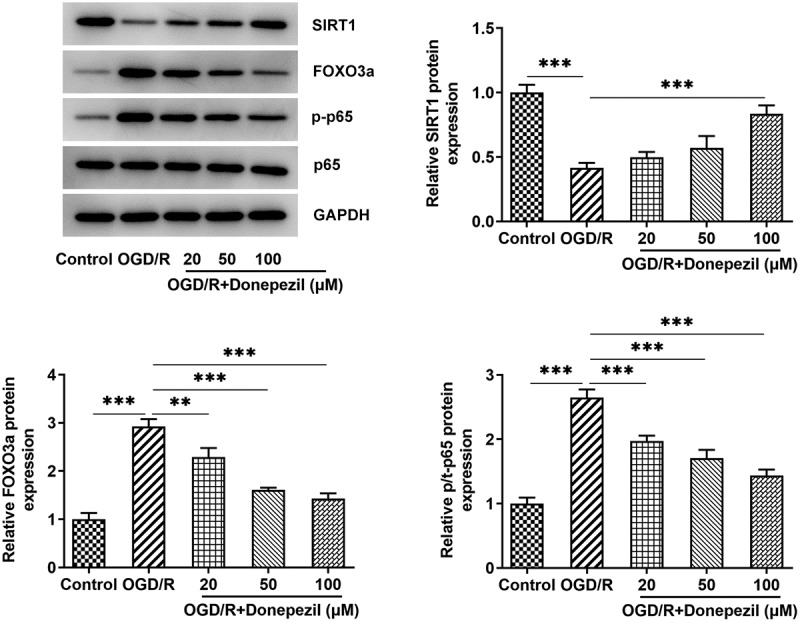
Donepezil regulates the SIRT1/FOXO3a/NF-κB signaling pathways. The expressions of SIRT1, FOXO3a, p-p65 and p65 were detected by the use of Western blot in OGD/R-induced HBMECs treated with donepezil (20, 50, 100 μM). Results are the mean ± SD. **P < 0.01, ***P < 0.001.

For further identifying the mechanism underlying the regulation of donepezil for OGD/R-induced HBMECs, SIRT1/FOXO3a/NF-κB signaling pathway was explored. The expressions of related proteins were examined in OGD/R-induced HBMECs treated with donepezil at the concentration of 20, 50, 100 μM. It was apparent from Figure 4 that Western blot detected decreased expression of SIRT1 and increased expressions of FOXO3a and p-p65 in the OGD/R group (vs Control), as well as elevated expression of SIRT1 and decreased expressions of FOXO3a and p-p65 in the OGD/R+ donepezil groups. While the expression of p65 in each group showed no difference. Together, these studies suggest that donepezil could exert a significant modulatory effect on SIRT1/FOXO3a/NF-κB signaling pathways. Subsequently, 100 μM donepezil was used for subsequent experiments due to its strong influence.

**Pretreatment with the SIRT1 inhibitor EX527 reversed the protective effect of donepezil on OGD/R-induced HBMECs**

**Figure 5.**
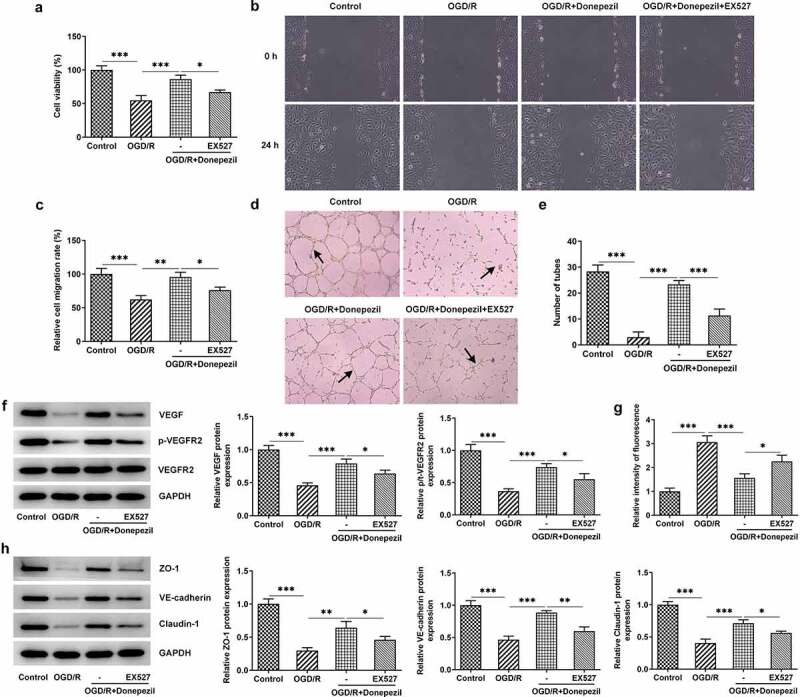
Pretreatment with the SIRT1 inhibitor EX527 reversed the protective effect of Donepezil on OGD/R-induced HBMECs. (a) Cell viability of HBMECs in the groups of OGD/R, OGD/R+ Donepezil and OGD/R+ Donepezil+EX527 was assessed with the help of CCK-8. (b-c) The migration capacity of HBMECs in the groups of OGD/R, OGD/R+ Donepezil and OGD/R+ Donepezil+EX527 was detected employing wound healing. (d-e) The angiogenesis of HBMECs was assayed in the groups of OGD/R, OGD/R+ Donepezil and OGD/R+ Donepezil+EX527 by means of tube formation. (f) The expressions of angiogenetic-related proteins VEGF, p-VEGFR2 and VEGFR2 in HBMECs were examined in the groups of OGD/R, OGD/R+ Donepezil and OGD/R+ Donepezil+EX527 by the way of Western blot. (g) The relative fluorescence intensity of HBMECs was measured in the groups of OGD/R, OGD/R+ Donepezil and OGD/R+ Donepezil+EX527 with the application of *in vitro* permeability test kit. (h) The expressions of related proteins ZO-1, VE-cadherin and Clarudin-1 in HBMECs were determined in the groups of OGD/R, OGD/R+ Donepezil and OGD/R+ Donepezil+EX527 employing Western blot. Results are the mean ± SD. *P < 0.05, **P < 0.01, ***P < 0.001.

For further confirmation of the protective effect of donepezil on HBMECs induced by OGD/R, we added the SIRT1 inhibitor EX527 to this group of experiments. From Figure 5(a) we can see that the addition of EX527 resulted in a successful decrease in cell viability of OGD/R-induced HBMECs treated with donepezil. Figure 5(b-c) showed a marked decline in the ability of cell migration in the OGD/R+ Donepezil+EX527 group. Likewise, EX527 markedly attenuated the ability of angiogenesis in OGD/R-induced HBMECs treated with donepezil (Figure 5(d-e)). Not only that, the expressions of angiogenesis-related proteins VEGF and p-VEGFR2 in OGD/R-induced HBMECs also decreased, but the expression of VEGFR2 kept the same after the addition of EX527 (Figure 5(f)). Subsequently, the impact of donepezil on the endothelial barrier function of HBMECs induced by OGD/R was assayed. It was found in Figure 5(g) that HBMECs showed a stronger fluorescence intensity in the OGD/R+ Donepezil+EX527 group that those in the OGD/R+ Donepezil group. Additionally, the expressions of ZO-1, VE-cadherin and Claudin-1 in OGD/R-induced HBMECs dropped by half after the addition of EX527 compared to the OGD/R+ Donepezil group (Figure 5(h)). Collectively, these studies outline a critical role for EX527 which can reverse the protective effect of donepezil on OGD/R-induced HBMECs.

## Discussion

Ischemic stroke is one of the leading causes of neurological morbidity and mortality worldwide [28]. Brain microvascular endothelial cells perform an essential role in brain ischemic injury. However, endothelial cell dysfunction is an early event that will induce lesions in the vessel wall, resulting in impaired ability of the endothelium to maintain vascular function and homeostasis [29]. Donepezil has been reported that it poses a protective effect on neuronal cells against cell injury [30]. In this paper, the protective effect of donepezil on OGD/R-induced injury of HBMECs was determined.

Donepezil is a reversible, noncompetitive acetylcholinesterase inhibitor that is mainly employed in the treatment of Alzheimer’s disease [16]. In this study, we used OGD/R to induce HBMECs to establish an in vitro model of brain ischemic cells and observed the effect of donepezil on HBMECs. Our experiments showed that donepezil-treated HBMECs did not exhibit significant changes in cell viability compared to the control group. While the cell viability decreased by OGD/R was successfully elevated by donepezil, and was highest at the donepezil concentration of 100 μM. These evidences implied that donepezil significantly protected the cell viability of OGD/R-induced HBMECs from being impaired.

Angiogenesis is the process of giving birth to a new vascular system, mainly capillaries [31]. In order to continuously meet the needs of their own blood supply, vascular endothelial cells secrete a variety of pro-vascular growth factors. Among them, vascular endothelial growth factor (VEGF) is a key regulator of angiogenesis and is also associated with cell proliferation and migration [32]. In addition, VEGF binds to its receptor VEGFR2, leading to the activation of VEGFR2 endocytosis and downstream signaling, promoting angiogenesis [33]. Donepezil can activate angiogenesis in patients with cardiovascular disease [34]. For instance, donepezil can improve ischemic muscle atrophy via activating angiomyogenic properties of satellite cells [35]. Donepezil also promotes angiogenesis in an ischemic hindlimb model [34]. In our experiments, the ability of cell migration reduced in the OGD/R model was significantly increased under the influence of donepezil. Depending on the number of vascular branches in the experiment, donepezil was also found to enhance angiogenesis of HBMECs. In addition, the levels of angiogenesis-related proteins VEGF and p-VEGFR2 were increased. These evidences strongly confirmed the promotive effect of donepezil on cell migration and angiogenesis in HBMECs induced by OGD/R.

In ischemic stroke, one of the pathophysiological features is the destruction of the BBB [36]. One of the most important features of BBB is its extremely strong intercellular adhesion, which is established by adhesion junctions (AJs) and tight junctions (TJs) between endothelial cells [37]. Tight junctions consist of transmembrane proteins, including occludin and claudins, which are attached to the cytoskeleton through interactions with ZO family proteins [38,39]. Additionally, the damage of BBB results in a marked increase in paracellular permeability at the level of the cerebral microvasculature [40,41]. Our experiments showed that the relative fluorescence intensity of endothelial barrier function decreased to a minimum in response to donepezil, while the levels of its associated proteins ZO-1, VE-cadherin, and Claudin-1 increased. All these evidences show that donepezil protects the endothelial barrier function from OGD/R-induced injury.

SIRT1, FOXO3a and NF-κB play important roles in the blood-brain barrier. Sirt1-Sirt3 axis regulates human blood-brain barrier permeability in response to ischemia [42]. Aralia taibaiensis could protect against I/R-induced brain cell injury through the Akt/SIRT1/FOXO3a pathway [43]. Melatonin exerts a protective role in the integrity and permeability of blood-brain barrier via suppressing matrix metalloproteinase-9 via the NOTCH3/NF-κB pathway [44]. To explore the effect of donepezil on these signaling pathways, we examined the levels of proteins associated with the signaling pathway. In our experiments, the elevated SIRT1 as well as the decreased FOXO3 and p-p65 levels suggest that donepezil also regulates the SIRT1/FOXO3a/NF-κB signaling pathways in OGD/R-induced HBMECs. To further investigate the effect of donepezil, we added EX527, an inhibitor of SIRT1, and the experimental results showed that cell viability was increased, cell migration and angiogenesis were diminished, and endothelial cell barrier function was impaired after EX527 pretreatment, all of which indicated that EX527 reversed the protective effect of donepezil on HBMEC. However, Cells in culture can behave very differently in the much more complex environment encountered in the brain, so in vitro experiments can only show that cells have the ability to behave as proposed, but they do not demonstrate that the mechanism is in effect in vivo. Furthermore, it is yet to be demonstrated clinically that improving endothelial function will be able to ameliorate reperfusion injury in stroke.

## Conclusion

On the basis of all the results studied here, donepezil was recognized as a key therapeutic agent of ischemic stroke. It can ameliorate OGD/R-induced brain microvascular endothelial cell dysfunction via the SIRT1/FOXO3a/NF-κB pathway, which is beneficial for the therapeutics of ischemic stroke.
